# Effects of total laryngectomy on olfactory function, health-related quality of life, and communication: a 3-year follow-up study

**DOI:** 10.1186/1472-6815-9-8

**Published:** 2009-07-29

**Authors:** Birgit Risberg-Berlin, Anna Rydén, Riitta Ylitalo Möller, Caterina Finizia

**Affiliations:** 1 Division of Logopedics and Phoniatrics, Sahlgrenska University Hospital, SE-413 45 Göteborg, Sweden; 2 Health Care Research Unit, Sahlgrenska University Hospital, SE-413 45 Göteborg, Sweden; 3 Karolinska Institute, Department of Otolaryngology B 53, Karolinska University Hospital Huddinge, SE-141 86 Stockholm, Sweden; 4 Department of Otolaryngology, Sahlgrenska University Hospital Mölndal, SE-431 80 Mölndal, Sweden

## Abstract

**Background:**

As total laryngectomy results in loss of airflow through the nose, one of the adverse effects for a majority of patients is the reduced or complete loss of olfactory function. However, with the introduction of a new method, the Nasal Airflow-Inducing Maneuver (NAIM), an important technique is available for laryngectomized patients to regain the ability to smell. The purpose of the present study was to assess changes in olfaction, health-related quality of life (HRQL) and communication 3 years after NAIM rehabilitation.

**Methods:**

18 patients (15 men and 3 women; mean age, 71 years) who had undergone laryngectomy and NAIM rehabilitation were followed longitudinally for 3 years. For comparison an age and gender matched control group with laryngeal cancer treated with radical radiotherapy was included. Olfactory function was assessed using the Questionnaire on Odor, Taste and Appetite and the Scandinavian Odor Identification Test. HRQL was assessed by: 1) the European Organization for Research and Treatment for cancer quality of life questionnaires; and 2) the Hospital Anxiety and Depression Scale. Communication was assessed by the Swedish Self-Evaluation of Communication Experiences after Laryngeal Cancer. Descriptive statistics with 95% confidence interval were calculated according to standard procedure. Changes over time as well as tests between pairs of study patients and control patients were analyzed with the Fisher nonparametric permutation test for matched pairs.

**Results:**

Thirty-six months after rehabilitation 14 of 18 laryngectomized patients (78%) were smellers. There were, with one exception (sleep disturbances), no clinically or statistically significant differences between the study and the control group considering HRQL and mental distress. However, statistical differences (p < 0.001) were found between the study and the control group concerning changes in communication.

**Conclusion:**

Olfactory training with NAIM should be integrated into the multidisciplinary rehabilitation program after total laryngectomy. Our study shows that patients who were successfully rehabilitated concerning olfaction and communication had an overall good HRQL and no mental distress. Moreover, the EORTC questionnaires should be complemented with more specific questionnaires when evaluating olfaction and communication in laryngectomized patients.

## Background

Laryngeal cancer is the most common malignant tumor of the upper aerodigestive tract. The clinical staging and the site of the larynx cancer will indicate different forms of treatment and consequently of rehabilitation with different impacts on health-related quality of life (HRQL) [1]. In advanced laryngeal cancer and cases of recurrence a total laryngectomy is mostly performed, resulting in a permanent disconnection of the upper and lower airways and a wide range of adverse effects. This anatomical change leads to loss of normal voice and deterioration in smell, taste and pulmonary function, with associated psychosocial problems affecting HRQL [2]. Despite the well known side effect of smell deterioration in laryngectomized patients effective rehabilitation in this area has only recently become available with the Nasal Airflow-Inducing Maneuver (NAIM), which so far has been evaluated in Holland and Sweden [2-5]. In the Swedish NAIM rehabilitation studies the sense of smell improved rapidly in 72% of the patients with anosmia or hyposmia after three NAIM rehabilitation sessions and the results persisted at 12 month follow-up [4,5].

During the last decade HRQL assessment has become an essential part of head and neck (H&N) cancer treatment evaluation and there has been a dramatic increase recently in the number of publications on HRQL following H&N cancer. These publications reflect the importance of the patient perspective as an outcome parameter in addition to survival, recurrence or physical function, where patient self-reported questionnaires are the mainstay of HRQL evaluation [6]. The most common self-completed measures used in H&N cancer patients are the European Organization for Research and Treatment of Cancer (EORTC) Quality of Life Questionnaires, proved to be statistically valid instruments [7,8], with a general part addressing all cancer patients (QLQ-C30) and a disease-specific H&N cancer module (QLO-H&N35). However, recent research has presented the need for adding more disease-specific questionnaires when assessing HRQL in laryngectomized patients in order to detect intervention-related changes over time in communication, respiration and smell [2,4,9,10].

By using the EORTC questionnaires in combination with more disease-specific questionnaires, such as the Questionnaire on Odor, Taste and Appetite (QOTA) and the Swedish Self-Evaluation of Communication Experiences after Laryngeal Cancer (S-SECEL) we primarily wanted to assess changes in olfaction, HRQL and communication during a 3-year period in laryngectomized patients who had received olfactory rehabilitation with the NAIM during 2002–2005. An additional aim was to compare differences in olfactory function, HRQL and communication between the laryngectomized study group and an age and gender matched control group of laryngeal cancer patients with preserved larynx.

## Methods

### Subjects and design

Of the 24 patients initially included in the rehabilitation program from 2002 through 2005, 18 were still alive and all of them were included in the present study [4,5]. The group consisted of 15 men and 3 women. Mean age was 71 years (range 57 – 83 years) and mean time since total laryngectomy was 10 years and ranged from 5–34 years. For comparison an age and gender matched control group of 18 patients with laryngeal cancer treated with radical radiotherapy, with preserved larynx and without any NAIM training were identified from the clinical records at the Department of Otolaryngology, Sahlgrenska University Hospital, Göteborg. All patients contacted agreed to participate and were included in the study. Mean age for the control group was 72 years (range 52 – 82 years) and mean time since radical radiotherapy was 10 years (range 2–31 years). Patients in both groups reported normal olfactory function before treatment of the H&N cancer and none of the patients had had any head trauma or severe respiratory infection resulting in olfactory deterioration. Additional health problems (cardiovascular disease) were reported by one laryngectomized patient and by two control patients. Patient characteristics are summarised in Table 1.

**Table 1:** Sociodemographic and clinical characteristics of study population and matched controls

**Characteristic**	**Study population** **(n = 18)**	**Matched controls** **(n = 18)**
Age, median years (range)	71 (57–83)	72 (52–82)
Last treatment mean years (range)	10.4 (5–34)	9.9 (2–31)
	**n (%)**	**n (%)**
**Sex**		
Female	3 (17%)	3 (17%)
Male	15 (83%)	15 (83%)
**Family situation**		
Married/Cohabitant	12 (67%)	14 (78%)
**Smoking**		
Never smoked	3 (17%)	1 (6%)
Stopped smoking > 1 year	14 (77%)	15 (83%)
Smoker	1 (6%)	2 (11%)
**Health problems**		
Cardiovascular disease	1 (6%)	2 (11%)
Pulmonar disease	0 (0%)	0 (0%)
Other malignancy	3 (17%)	0 (0%)
**Communication**		
Alaryngeal voice		
Prostheses	10 (56%)	
Esophageal	2 (11%)	
Electrolarynx	5 (28%)	
Pseudowhisper	1 (5%)	
Laryngeal voice		18 (100%)

For the laryngectomized patients (study group) data were collected at baseline (i.e. before NAIM rehabilitation), and at 6 and 36 months follow-up sessions after initial rehabilitation in order to register changes in olfaction, HRQL and communication. One patient was not followed-up at 6 month due to concomitant disease. The control group was only examined once. EORTC QLQ-C30 results from the study group were also compared to those of a reference group, i.e. a random sample of 234 men aged 70–79 in the Swedish population drawn from a population-based registry (SEMA) including all Swedish inhabitants born between 1918 and 1979 [11].

The study was conducted in accordance with the Declaration of Helsinki and was approved by the Ethical Board of the Sahlgrenska University Hospital, Göteborg, Sweden.

### Olfaction rehabilitation

During the primary rehabilitation period (2002–2005), speech-language pathologists (including the author B R-B) trained patients in the study group in the use of NAIM, which creates a negative pressure in the oral cavity and oropharynx to induce orthonasal airflow, thus enabling odorous substances to reach the olfactory epithelium. Patients were instructed to make an extended yawning by lowering the jaw, floor of mouth, tongue, base of the tongue, and soft palate, while the lips are closed. Three intervention sessions were performed during 6 weeks. Patients were instructed to actively use the maneuver as frequently as possible and try to integrate it into daily life after the primary rehabilitation period and repetition at the 6-month follow-up [4,5].

### Examination

#### The Scandinavian Odor Identification Test (SOIT)

Olfactory function was tested with the Scandinavian Odor Identification Test (SOIT) [12]. This test has age and gender related cut-off scores and categorizes the sense of smell in 3 diagnoses: normosmia, hyposmia, or anosmia. The cut-off scores used in this study for age group 55 to 74 years were ≤7 points for anosmia, 8–10 for hyposmia and 11–16 for normosmia. On the basis of performance on the SOIT, patients were categorized as smellers or non-smellers. Smellers were patients having a diagnosis of functional hyposmia or normosmia and non-smellers were patients with anosmia.

#### Semi-structured interview

A semi-structured interview including questions on smell and taste was conducted at each session. Calculated scale scores ranged from 0 to 100, where 0 corresponded with "very bad" and 100 with "very good". In addition, the active use of NAIM was asked for. This procedure is described in more detail in Risberg-Berlin et al 2006 [5].

#### Questionnaires

Five structured self-reported questionnaires were used for olfactory assessment, HRQL and communication: 1) Questions on Odor, Taste and Appetite (QOTA); 2) The European Organization for Research and Treatment of Cancer (EORTC) Quality of Life Core Questionnaire (QLQ-C30); 3) The EORTC Quality of Life Head and Neck Module (EORTC QLQ-H&N35); 4) The Hospital Anxiety and Depression Scale (HADS); and 5) The Swedish Self-Evaluation of Communication Experiences after Laryngeal Cancer questionnaire (S-SECEL). Completion of the questionnaires including olfactory testing took approximately 2 hours.

##### The Questionnaire on Odor, Taste and Appetite (QOTA)

QOTA have demonstrated satisfactory validity and reliability and consists of several multiple-choice questions addressing both the pre- and post treatment situations as well as the present situation. Questions are divided into 5 scales: 1) present taste perception (8 items; score range 8–40); 2) appetite (6 items; score range 6–30); 3) present odor perception (POPS), (3 items; score range 3–15); 4) present odor perception compared with past (3 items; score range 3–15); and 5) daily feelings of hunger (9 items; score range 9–45). A low score indicates poor function or deterioration of these functions compared to pre-treatment scores. Conversely, a high score indicates good function or improvement in these functions [13].

##### The European Organization for Research and Treatment of Cancer (EORTC QLQ-C30 and EORTC QLQ-H&N35)

EORTC QLQ-C30. This 30-item questionnaire is a widely used, cancer-specific, patient-based measure designed for self-administration. The QLQ-C30 was used to assess patients' HRQL, including general physical and psychosocial functioning and symptoms [7]. To address symptoms associated specifically with head and neck cancer and its treatment we used the complementary 35-item EORTC H&N Module (QLQ-H&N35) [8]. Both questionnaires have demonstrated satisfactory reliability and validity when tested in large, cross-cultural samples of cancer and H&N cancer patients. Calculated scores for C30 and H&N35 range from 0–100, with 100 indicating maximum functioning (functioning scales and global HRQL) or worst symptoms (symptom scales and items) [8]. HRQL scores were calculated according to the QLQ-C30 scoring manual [14] and a difference (Δ) of 10 points or more was regarded as clinically significant [15].

##### The Hospital Anxiety and Depression Scale (HADS)

The HAD scale measures general distress [16]. It consists of 14 items on a four-point response scale that are summed up to separate scores on anxiety and depression. Each person is also grouped according to a clinically tested classification of psychiatric morbidity. A scale score of less than 8 is in the normal range, a score 8 to 10 indicates a possible case, and a score greater than 10 indicates a probable mood disorder. The Swedish version has been documented in several studies [9,17].

##### The Swedish Self-Evaluation of Communication Experiences after Laryngeal Cancer (S-SECEL)

The original Self-Evaluation of Communication Experiences after Laryngectomy (SECEL) was developed to assess communication dysfunction in patients with laryngectomies and has demonstrated satisfactory psychometric properties [18]. The Swedish version (S-SECEL) was adapted for use in patients receiving different treatments for laryngeal cancer and has proved reliable and shown both convergent and discriminant validity and satisfactory internal consistency [9,19]. S-SECEL consists of 35 items addressing communication experiences and dysfunction in patients receiving different treatments for laryngeal cancer. Thirty-four of the items are aggregated into 3 subscales to measure general (5 items; score range 0–15), environmental (14 items; score range 0–42) and attitudinal (15 items; score range 0–45) communication experiences, as well as a total scale (score range 0–102). Each item is rated on a 4-point scale ranging from 0 (never) to 3 (always) and scoring of subscales and a total scale is carried out by simple addition (0–102 p). A higher score indicates greater perceived communication dysfunction. Item no. 35: "Do you talk as much now as before your laryngeal cancer?" is answered by three response categories (Yes/More/Less), and is not included in the scoring system.

### Statistical analysis

Descriptive statistics with 95% confidence interval (CI) were calculated according to standard procedure. Changes over time as well as tests between pairs of study patients and control patients were analyzed with the Fisher nonparametric permutation test for matched pairs. When estimating clinical significance in the EORTC questionnaires, changes over time within the study group and differences in mean scores between groups were assessed according to recommendations by Osoba, where a difference in HRQL scores of 10 points or more is regarded clinically significant [15]. All tests were 2-tailed and conducted at a 5% significance level. [20].

## Results

### Subjects

There were no significant differences between the study and the control group regarding radiation dose given (Gray) or socio-demographic and clinical characteristics (Table 1), with the exception of mode of communication.

### Olfaction

#### SOIT score and categories

Results of the olfactory function over time according to SOIT scores and categories are presented in Table 2. At baseline 11 patients (61%) were categorized as non-smellers, i.e. had anosmia, while 7 patients (39%) were smellers; normosmia (n = 5) and hyposmia (n = 2). In two of the non-smellers (anosmia) and four of the smellers (normosmia) the SOIT category did not change over time. At 6-month follow-up 7 of the 10 non-smellers (70%) became smellers (hyposmia). One patient could not be examined at this time point due to concomitant disease.

**Table 2:** Score changes from pretreatment (baseline) to 36 months posttreatment in study population and control patients

	**Mean (95% CI) Score**						
**Characteristic**	**Study population** **(n = 18)**					**Controls** **(n = 18)**	**p study/control**
	**Baseline**	**6 mo**	**p**	**36 mo**	**p**		

SOIT score^a^	7.2 *(5.1–9.2)*	9.4 *(7.6–11.3)*	.03	9.5 *(7.7–11.3)*	.003	13.7 *(13.1–14.3)*	*< .001*
Patients' self-estimation^b^							
Present olfaction	25.9 (11.7–40.2)	63.7 *(52.3–75.2)*	< .001	55.6 *(42.5–68.6)*	< .001	76.5 *(65.5–87.4)*	*.007*
Present gustation	67.6 *(51.2–84.0)*	81.4 *(69.7–93.1)*	.04	78.7 *(63.7–93.7)*	.20	83.3 *(76.6–90.1)*	*.36*
QOTA^c^							
Taste	26.3 *(24.3–28.4)*	28.1 *(25.9–30.3)*	*.01*	27.2 *(24.7–29.7)*	.53	29.5 *(28.2–30.8)*	*.09*
Appetite	22.4 *(20.7–24.1)*	22.8 *(21.0–24.7)*	*.26*	23.1 *(21.5–24.6)*	*.51*	23.4 *(22.3–24.6)*	*.72*
POPS	7.9 *(6.0–9.8)*	9.4 *(8.4–10.5)*	*.02*	9.6 *(8.2–10.9)*	.03	12.8 *(11.8–13.8)*	*< .001*
Present sense of smell compared with pretreatment	5.4 *(4.3–6.6)*	7.4 *(5.6–9.2)*	.03	6.3 *(4.9–7.8)*	.06	10.0 *(9.1–10.9)*	*< .001*
Daily feelings of hunger	32.0 *(30.2–33.8)*	32.1 *(30.5–33.7)*	.79	32.1 *(29.8–34.4)*	.97	33.6 *(31.8–35.4)*	.67

Abbreviations: CI, confidence interval;

^a^SOIT, Scandinavian Odor-Identification Test. Score range, 11–16 for normosmia, 8–10 for hyposmia, and ≤ 7 for anosmia;

^b^Patients' self-estimation; Score range, 0–100, where 0 corresponds to worst perceived smell and taste;

^c ^QOTA = Questionnaire on Olfaction, Taste and Appetite; Taste, 8 items, score range per item, 8 to 40; Appetite, 6 items, score range per item, 6 to 30; POPS, 3 items, score range per item, 3 to 15; Present sense of smell vs. preoperative, 3 items, score range per item 3 to 15; Daily feelings of hunger, 9 items, score range per item, 9 to 45. A low score indicates bad function or deterioration of these compared with the pretreatment situation.

At 36-month follow-up 14 of 18 patients (78%) were categorized as smellers; normosmia (n = 8) and hyposmia (n = 6), while 4 patients (22%) still were non-smellers (anosmia).

The SOIT score improvement over time within the study group was statistically significant (p = 0.029, p = 0.003 respectively).

#### Patients' self-estimation and QOTA

According to patients' self-estimation of smell significant improvements in olfactory function compared to baseline were seen at 6 and 36 month follow-up (p < 0.001), and for taste at 6 month follow-up (p = 0.039). A significant improvement over time was also found according to the QOTA scales "Present sense of smell", "Appetite" (6 and 36 month) and "Taste" (6 month).

#### Use of the NAIM

At the 36-month follow-up, 12 of 18 patients (67%) were active users of the olfactory technique and used it "automatically", i.e. on a daily basis. Of the 6 patients not using NAIM, 2 were smellers and 4 non-smellers.

#### Study group vs. control group

The matched controls were all smellers (normosmia n = 18) and scored significantly better than the study group according to SOIT (p < 0.001), Table 2. The QOTA scales "Present sense of smell", "Appetite" and "Present sense of smell compared to before treatment" also showed significantly better results in the controls than in the study group (p < 0.001), Table 2.

### Questionnaires

#### EORTC

##### QLQ-C30

EORTC OLQ-C30 results are presented in Table 3. No significant within-group differences were found for the study group over time. Additionally, when comparing the study group with the controls no significant between-group differences were found with the exception for the symptom "Sleep disturbances" (Δ 13) in favour of the controls, i.e. less disturbed sleep.

**Table 3:** Mean values (95% CI) of EORTC QLQ-C30 scores from pretreatment (baseline) to 36 months posttreatment in study- and control patients

	**Mean (95% CI) Score**							
**Scale name**	**Study population** **(n = 18)**					**Controls** **(n = 18)**	**p study/control**	**Norm data^a^**
	**Baseline**	**6 mo**	**p**	**36 mo**	**p**			

**Functional scales**								
Physical	88.9 *(84.2–93.6)*	89.4 *(83.1–95.7)*	.52	85.9 *(79.5–92.4)*	.31	91.1 *(86.3–95.9)*	.07	*81.6*
Role	93.5 *(86.5–100.6)*	85.3 *(76.8–93.8)*	.16	86.1 *(72.1–100.1)*	.28	90.7 *(83.1–98.4)*	.69	*82.6*
Emotional	94.9 *(90.4–99.4)*	90.2 *(84.3–96.1)*	.09	91.2 *(85.6–96.8)*	.18	92.1 *(85.0–99.3)*	.85	*88.2*
Cognitive	94.4 *(90.4–98.5)*	97.1 *(93.7–100.4)*	.25	98.2 *(94.2–102.1)*	.22	92.6 *(85.5–99.7)*	.06	*85.2*
Social	94.4 *(88.1–100.9)*	90.2 *(80.6–99.8)*	.53	85.2 *(73.2–97.2)*	.18	90.7 *(83.1–98.4)*	.44	*89.1*
Global QLQ	85.7 *(78.9–92.4)*	79.4 *(69.1–89.7)*	.27	80.6 *(72.2–89.0)*	.12	82.9 *(76.4–89.3)*	.54	*76.4*
**Symptom scales**								
Fatigue	9.9 *(3.6–16.1)*	15.0 *(6.5–23.6)*	.11	14.8 *(7.7–21.9)*	.14	14.8 *(8.8–20.8)*	.84	*21.5*
Nausea and vomiting	0.9 *(-1.0–2.9)*	1.0 *(-1.1–3.1)*	.98	1.9 *(-0.8–4.5)*	.93	0.9 *(-1.0–2.9)*	^b^	*2.5*
Pain	5.6 *(-0.8–11.9)*	3.9 *(0.2–7.7)*	.75	13.9 *(3.5–24.2)*	.11	13.0 *(4.2–21.8)*	.88	*19.2*
**Symptom single items**								
Dyspnoea	29.6 *(20.0–39.3)*	33.3 *(24.8–41.9)*	.25	27.8 *(17.5–38.0)*	.75	24.1 *(14.6–33.6)*	.38	*23.7*
Sleep disturbances	22.2 *(5.2–39.3)*	19.6 *(6.0–33.2)*	.95	20.4 *(6.3–34.5)*	.95	7.4 *(-1.7–16.5)*	.20	*11.8*
Appetite loss	1.9 *(-2.1–5.8)*	2.0 (-2.2–6.1)	^b^	0.0 *(..)*	^b^	1.9 *(-2.1–5.8)*	^b^	*2.7*
Constipation	5.6 *(-3.0–14.1)*	7.8 *(-1.8–17.5)*	.50	7.4 *(0.3–14.5)*	.50	7.4 *(0.3–14.5)*	.72	*6.7*
Diarrhoea	0.0 *(..)*	3.9 *(-1.8–9.6)*	^d^	3.7 *(-1.7–9.1)*	^d^	5.6 *(-0.8–11.9)*	>.99	*4.2*
Financial problems	1.9 *(-2.1–5.8)*	0.0 *(..)*	^b^	0.0 *(..)*	^c^	0.0 *(..)*	>.99	*5.4*

Abbreviations: CI, confidence interval; EORTC, The European Organization for Research and Treatment of Cancer Core Questionnaire (C30) scale: range per scale: 0–100 where 100 corespond to maximum functioning. Symptom scales and items; where 100 correspond to worst symptoms. Clinically significant change, i.e. a change of ≥10 points.

^a ^Reference values for EORTC QLQ-C30 for 234 men aged 70–79 in the Swedish population drawn from a population-based registry (SEMA) including all Swedish inhabitants born between 1918 and 1979 [11].

^b ^The number of observations after zeros is removed as it is 1 so there is no use calculating the p-value

^c ^The number of observations after zeros is removed as it is 0 so there is no use calculating the p-value

^d ^The number of observations after zeros is removed as it is 2 so there is no use calculating the p-value

When compared with the reference group, the study group scored higher on 9 of the 15 scales and single items in EORTC C30 [11]. However, these differences were not clinically significant.

##### QLQ-H&N35

Table 4 shows results of the EORTC QLQ-H&N35. In general, score values were stable for the study population across all measurement points, except for a clinically significant deterioration for sexuality (Δ 10).

**Table 4:** Mean values (95% CI) of EORTC QLQ-H&N35 scores from pretreatment (baseline) to 36 months posttreatment in study- and control patients

	**Mean (95% CI) Score**						
**Scale name**	**Study population** **(n = 18)**					**Controls** **(n = 18)**	**p study/control**
	**Baseline**	**6 mo**	**p**	**36 mo**	**p**		

**Symptom scales**							
Pain	0.0 *(..)*	5.8 *(-2.0–13.7)*	.25	6.5 *(-0.4–13.4)*	.06	3.2 *(-1.1–7.5)*	*.44*
Swallowing	8.8 (0.1–17.5)	4.9 *(0.6–9.2)*	>.99	13.9 *(2.2–25.6)*	.34	8.3 *(-0.7–17.3)*	*.18*
Senses	35.2 *(21.9–48.5)*	31.4 *(18.9–43.8)*	.67	31.5 *(17.9–45.1)*	.62	7.4 *(-1.2–16.0)*	*.002*
Speech	13.6 *(6.1–21.1)*	9.2 *(4.5–13.8)*	.36	14.8 *(6.3–23.3)*	.70	13.6 *(7.4–19.7)*	*.76*
Social eating	6.0 *(0.7–11.3)*	1.5 *(-0.2–3.2)*	.16	13.9 *(-0.2–28.0)*	.21	6.9 *(-4.8–18.7)*	*.16*
Social contact	5.9 *(1.7–10.2)*	2.8 *(-0.9–6.4)*	.25	5.2 *(0.4–10.0)*	.88	3.0 *(-1.9–7.8)*	*.50*
Sexuality	11.1 *(3.1–19.2)*	10.8 *(1.2–20.3)*	.88	22.6 *(7.1–38.0)*	.13	39.8 *(21.8–57.8)*	*.02*
**Symptom single items**							
Teeth	9.3 *(-4.4–23.0)*	2.0 *(-2.2–6.1)*	^b^	9.3 *(1.6–16.9)*	>.99	7.4 *(-1.7–16.5)*	.88
Opening mouth	1.9 *(-2.1–5.8)*	3.9 *(-1.8–9.6)*	^a^	9.3 *(-3.2–21.7)*	.25	0.0 *(..)*	.25
Dry mouth	11.1 *(1.3–21.0)*	11.8 *(3.3–20.2)*	.75	16.7 *(2.5–30.9)*	.38	13.0 *(1.4–24.5)*	.61
Sticky saliva	14.8 *(3.1–26.5)*	9.8 *(1.8–17.9)*	.73	22.2 *(5.2–39.3)*	.36	18.5 *(5.5–31.5)*	*.52*
Coughing	16.7 *(6.4–26.9)*	17.7 *(8.8–26.5)*	.72	16.7 *(6.4–26.9)*	.72	20.4 *(7.5–33.3)*	*.62*
Felt ill	3.7 *(-4.1–11.5)*	5.9 *(-0.9–12.6)*	>.99	3.7 *(-1.7–9.1)*	^b^	5.6 *(-3.0–14.1)*	*.88*
**Senses scale separated on item level**							
Problems with olfaction	55.6 *(35.1–76.1)*	43.1 *(26.3–60.0)*	.32	44.4 *(26.5–62.4)*	.30	13.0 *(-2.2–28.2)*	*.004*
Problems with taste	14.8 *(1.8–27.8)*	19.6 *(4.7–34.5)*	.38	17.7 *(1.5–33.8)*	.68	1.9 *(-2.1–5.8)*	*.06*

Abbreviations: CI, confidence interval; EORTC, The European Organization for Research and Treatment of Cancer Head & Neck Module (H&N35) range per symptom scale: 0–100, where 100 correspond to worst symptoms. Clinically significant change, i.e. a change of ≥ 10 points.

^a ^The number of observations after zeros is removed as it is 1 so there is no use calculating the p-value

^b ^The number of observations after zeros is removed as it is 2 so there is no use calculating the p-value

However, when the study group was divided into smellers and non-smellers (data not shown) a clinically significant difference was found in the following scales and items: Senses (Δ 20); Speech (Δ 10); Dry mouth (Δ 11); and Sticky saliva (Δ 36), all in favour of the smellers.

When comparing the study group with the controls both clinically and statistically significant differences were found in Senses scale (Δ 24, p = 0.002; less disturbed in the controls) and for Sexuality (Δ 17, p = 0.016; less disturbed in the study group).

#### HADS

At all measurement points score values were stable and low for the study group. At follow-up (36 months) the study group reported possible/probable anxiety or depression disorder in 0% and 6%, respectively. Corresponding values for the controls were 11% and 0% for possible/probable anxiety or depression.

#### S-SECEL

No significant differences in S-SECEL scores were shown for the study group over time and most communication problems were found in the Environmental scale, Table 5.

**Table 5:** Mean values (95% CI) for S-SECEL total and subscale scores from pretreatment (baseline) to 36 months posttreatment in study- and control patients

	**Mean (95% CI) Score**						
**Scale name**	**Study population** **(n = 18)**					**Controls** **(n = 18)**	**P study/control**
	**Baseline**	**6 mo**	**p**	**36 mo**	**p**		

General^a^	4.2 *(3.0–5.5)*	4.8 *(3.4–6.3)*	.57	4.3 *(3.2–5.5)*	.91	3.8 *(2.8–4.9)*	*.55*
Environment^a^	13.4 *(11.0–15.9)*	12.4 *(9.5–15.4)*	.48	14.8 *(10.7–18.9)*	.57	7.6 *(4.8–10.4)*	*.006*
Attitudinal^a^	7.4 *(5.2–9.6)*	7.8 *(4.3–11.4)*	.88	10.7 *(6.8–14.7)*	.17	1.7 *(0.5–2.8)*	*< .001*
Total^a^	25.1 *(20.2–29.9)*	25.1 *(19.1–31.2)*	> .99	29.8 *(21.7–37.9)*	.27	13.1 *(9.2–16.9)*	*< .001*

Abbreviations: CI, confidence interval;

S-SECEL, The Swedish Self-Evaluation of Communication Experiences after Laryngeal Cancer;

^a ^Min-max: 0–15 (general), 0–42 (environmental), 0–45 (attitudinal), and 0–102 (total). A low value indicates better communication

When the study group was divided into smellers and non-smellers, results from baseline, 6 and 36 month follow-up showed that non-smellers deteriorated over time according to total S-SECEL mean values (24.1; 26.7 and 35.1), whereas smellers improved (26.6; 22,6 and 21,6).

The study group as a whole reported more problems with speech and communication than the controls. Statistical significance was noted for all scales with the exception of the General subscale. The largest difference between the groups was found in the Attitudinal subscale.

## Discussion

The present study has a longitudinal design and is to our knowledge the first study to assess olfactory rehabilitation with NAIM and HRQL in laryngectomized patients over a period of 3 years.

Important findings in this study were the continued improvement of olfactory function after NAIM rehabilitation both according to SOIT and patients' self-estimation of smell during a 3-year period. This study confirms our previous results, i.e. the importance of follow-up and repetition of the NAIM [4] to make the technique a patient automatism with integration in daily life, resulting in positive effects for patients concerning for example food and cooking, odors in nature and personal hygiene. Six patients (2 smellers and 4 non-smellers) did not use the technique regularly. Among these patients the smellers reported good olfaction. One was an esophageal speaker and the other one had found his own smelling technique, similar to the NAIM. Reasons for the non-smellers (n = 4) not to use the NAIM was finding it too conspicuous in public, difficult to apply, bad motivation to learn or poor general health.

Contrary to what could be expected, our laryngectomized patients reported HRQL scores comparable to those of the controls, and better HRQL than reported in previous studies in laryngectomized patients [1,17,21]. This might be explained by several factors. The majority (83%) of the study group was successfully rehabilitated and all patients had completed their therapy concerning communication, breathing and swallowing. The time interval between laryngectomy or radiotherapy and the start of our study also allowed patients' recovery from anatomic and functional alterations independent of treatment alternative. In a study by Birkhaug et al. a positive association was reported between level of activity within the Norwegian Society of Laryngectomies and HRQL scores [21]. Eleven of our study patients (61%) were members of a patient organization, which might have influenced the HRQL. Furthermore, somewhat surprisingly, both the study and control group reported better HRQL when compared to a reference group including data from a general age-matched Swedish normal population sample [11].

In the present study only few differences were found in the EORTC QLQ-H&N35 module between the laryngectomized study group and the control group treated with radiotherapy, results also confirmed in other studies [1,17,22]. However, an interesting finding in the study group concerns one of the two questions in the Senses scale, "Problems with smell", that displayed a clinically significant change after NAIM rehabilitation, whereas the Senses scale (also including "Problems with taste") did not. Bindewald et al. [23] suggested that these two items should be analysed separately since the scale has previously shown low internal consistency [8,23]. We agree with Bindewald et al. as this would be especially relevant in laryngectomized patients due to the varying meaning of the questions for these patients. Furthermore, a divided Senses scale may have increased the sensitivity to capture olfaction improvement over time in our study group. An alternative could be to use the QOTA, a questionnaire with more items on olfaction, taste and appetite that captured significant changes over time concerning important aspects for the study patients. However, this questionnaire consists of 29 questions and the amount of patient-reported questions should always be carefully considered when deciding which questionnaires to use in research studies as well as in clinical settings.

From our results it could be argued that the QLQ-H&N35 questionnaire also lacks in sensitivity regarding the Speech scale. In the study group, with alaryngeal communication, a higher score on this scale could be expected, i.e. more speech and communication problems, but instead they scored equally to the control group with laryngeal communication (mean EORTC values of 14.8 and 13.6 respectively). However, a significant difference between the study and control group clearly indicated that the laryngectomized patients perceived greater speech and communication problems. The poor sensitivity of the speech scale in the QLQ-H&N35 module are findings in line with those of several other studies, suggesting the need to use additional questionnaires to capture changes over time for specific symptoms such as communication, respiratory and smell problems, especially in laryngectomized patients [1,9,10].

Another communication finding was that according to the total S-SECEL score patients categorized as smellers also seemed to judge themselves as more successfully rehabilitated concerning communication then non-smellers did. It might be suggested that there is a connection between good voice production and olfactory technique in laryngectomized patients.

Neither the study, nor the control group reported any anxiety or depression, and these findings are in line with other studies with laryngeal cancer patients [9,10]. Only one of the study patients exceeded the cut-off value for depression at 36-month follow-up which might be related to problems with swallowing (use of gastric feeding tube) and communication (pseudo whisper).

A limitation in our study is the small number of patients since this did not allow for comparisons between gender or time since start/end of treatment. As the majority of our patients were well rehabilitated (communication, breathing, swallowing and social life) and highly motivated to participate in the study the high HRQL results may be due to selection bias. However, the 11 patients in our catchment area not participating in the NAIM rehabilitation study, had better self-estimated olfaction and similar HRQL results at study start than the study group, indicating a minor risk of a selection bias [5]. An additional explanation for the different results may be coping strategies and psycho-social factors of H&N cancer patients and their adaptation to living with the disease with time [24,25].

## Conclusion

Our study shows that laryngectomized patients who were successfully rehabilitated concerning smell and communication had an overall good HRQL and no mental distress.

We recommend that olfactory testing and training with NAIM should be integrated into the multidisciplinary rehabilitation program after total laryngectomy.

Furthermore, our results show the use of additional validated survey instruments to the EORTC questionnaires when evaluating specific functions such as olfaction and communication in laryngectomized patients.

## Competing interests

The authors declare that they have no competing interests.

## Authors' contributions

The individual contributions of the authors to the manuscript: 1) BRB, AR and CF have made substantial contributions to conception and design, acquisition of data, analysis and interpretation of data; 2) BRB and CF has been involved in drafting the manuscript and AR and RYM in revising it critically for important intellectual content; and 3) All authors have read and approved the version to be published.

## Pre-publication history

The pre-publication history for this paper can be accessed here:


